# A growth area: A review of the value of clinical studies of child growth for palaeopathology

**DOI:** 10.1093/emph/eoac005

**Published:** 2022-02-08

**Authors:** Sarah-Louise Decrausaz, Michelle E Cameron

**Affiliations:** 1 Department of Anthropology, University of Victoria, Cornett Building, Victoria, BC V8P 5C2, Canada; 2 Department of Anthropology, University of Toronto, 19 Ursula Franklin Street, Toronto, ON M5S 2S2, Canada

**Keywords:** growth, childhood, adolescence, palaeopathology, human biology

## Abstract

Studies of living children demonstrate that early life stress impacts linear growth outcomes. Stresses affecting linear growth may also impact later life health outcomes, including increased cardiometabolic disease risk. Palaeopathologists also assess the growth of children recovered from bioarchaeological contexts. Early life stresses are inferred to affect linear growth outcomes, and measurements of skeletal linear dimensions alongside other bioarchaeological information may indicate the types of challenges faced by past groups. In clinical settings, the impacts of stress on growing children are typically measured by examining height. Palaeopathologists are limited to examining bone dimensions directly and must grapple with incomplete pictures of childhood experiences that may affect growth. Palaeopathologists may use clinical growth studies to inform observations among past children; however, there may be issues with this approach. Here, we review the relationship between contemporary and palaeopathological studies of child and adolescent growth. We identify approaches to help bridge the gap between palaeopathological and biomedical growth studies. We advocate for: the creation of bone-specific growth reference information using medical imaging and greater examination of limb proportions; the inclusion of children from different global regions and life circumstances in contemporary bone growth studies; and greater collaboration and dialogue between palaeopathologists and clinicians as new studies are designed to assess linear growth past and present. We advocate for building stronger bridges between these fields to improve interpretations of growth patterns across human history and to potentially improve interventions for children living and growing today.

## Lay Summary

Studies of living children demonstrate that early life stress impacts linear growth. Stresses affecting linear growth may also impact later life health, including cardiometabolic disease risk. Palaeopathologists also investigate if children and adolescents recovered from bioarchaeological contexts experienced growth disruptions due to early life challenges. In clinical settings, the impacts of stress on growing children are typically measured by examining height. Palaeopathologists are limited to examining bone dimensions directly and must grapple with incomplete pictures of childhood experiences that may affect growth. Here, we review the relationship between contemporary and palaeopathological studies of child and adolescent growth. We advocate for: the creation of bone-specific growth reference information using medical imaging and greater examination of limb proportions; the inclusion of children from different global regions and life circumstances in contemporary bone growth studies; and greater collaboration and dialogue between palaeopathologists and clinicians as new studies are designed to assess linear growth past and present. These steps may improve interpretations of growth patterns across human history and interventions for children living and growing today.

## INTRODUCTION

Growth among infants, children and adolescents is an important component of ontogeny. Growth refers to changes in size, which are variable in absolute magnitude (distance) and rate of change (velocity), and result in variation in adult size [1]. A full set of definitions for growth, maturation, auxology and development as used in this article are available in Table 1. Growth is heritable but also represents the interaction of genetic and environmental factors in the acquisition of adult phenotypes [6–16]. Environmental factors may include, for example, nutritional status [10, 13], infectious disease loads [17–19] and psychosocial stresses [20].

**Table 1.** List of terminology used in this article to define age categories and concepts relating to growth

Term	Definition in this article
Infants	Individuals between two and 36 months [2]
Children	Individuals between four to 9 years
Adolescents	Individuals between 10 and 24 years [3]
Adults	Individuals above 25 years
Auxology	The study or the science of human growth and development [4]. Auxology makes use of empirical evidence, particularly change of size of body parts and the body overall, in relation to an individual’s known birth date [1].
Growth^a^	A dynamic term indicating change per unit time, often including a quantitative increase in size or mass over a specific unit of time, such as months or years. In auxology, growth includes the consideration of age, size and the changes of size with age [1].
Development^a^	Changes to the soft tissues (for example, increased localized adiposity with hormonal changes) with age over time [1].
Maturation	Functional changes which occur with age over time in a definable pattern, moving from an immature status to a mature status. Functional changes associated with maturation occur throughout the body and include dental maturation, sexual maturation (such as menarche and spermache), skeletal maturation and somatic maturation [1].
Distance curve of growth	One type of curve representing growth on a growth chart. The distance curve shows the amount of growth from one year to the next as an individual grows [2].
Velocity curve of growth	A type of curve representing growth on a growth chart. Growth velocity curves show the rate of growth during any one year [2].
Peak height velocity	The period of time in which an individual experiences the fastest increase in height, most often during adolescence [5].

The first column shows the term used in this article, and the second column shows a complete definition of the term, including relevant citations, as used in this article.

a See Ref. [1] for a full discussion of differences in the use of these terms in auxology compared to palaeoanthropological studies.

In this article, we focus on linear growth among children and adolescents across development. Intrauterine and infant growth are also impacted by genetic, endocrine and metabolic factors, potentially resulting in intrauterine growth restriction (IUGR) and compromises in postnatal growth and development [21, 22]. However, discussion of IUGR and limitations in infancy are beyond the scope of the present article. We appreciate the important role of environmental influences during the intrauterine and infant growth periods; however, we argue that growth among children and adolescents has received comparatively less attention in recent years.

Investigating what constitutes a ‘healthy’ growth pattern and the factors that cause individuals to deviate from this pattern is essential for child and population health. Healthy growth and development may be defined as the interaction of physical, mental, emotional and social well-being during ontogeny [23]; however, health is a difficult concept to define [24]. Given this difficulty, there is an emphasis on quantifying ‘normal’ growth patterns and highlighting instances where growth deviates from these trajectories. We acknowledge that normal growth patterns, whilst analytically useful, may obscure important variability and be subject to bias due to the historical exclusion of certain bodies [25]. However, the evaluation of population-level patterns and their average tendencies helps to identify expected patterns of child and adolescent growth, instances where growth may falter, and the factors that may cause growth to falter [26].

Growth patterns may be altered through developmental plasticity and the canalization of essential organs [27–29]. Developmental plasticity is the moderation of phenotypes due to environmental conditions during ontogeny [28]. This adaptive response may occur through canalization, where the growth and development of traits varies to compensate for environmental circumstances. This may involve the targeted growth associated with catch-up growth, or the compensatory growth of certain key organs, such as the heart or brain, at the expense of other systems such as the skeleton [27]. Plasticity has also been conceptualized with the thrifty phenotype hypothesis, where nutritional constraints compromise size acquisition to preserve metabolic function [15, 28]. The skeleton is ideal for assessing if developmental plasticity has affected growth outcomes. Resources may be allocated away from skeletal development to preserve the function of essential organs in stressful periods [15, 30]. Measurements closely tied to skeletal development, such as height, may indicate if an individual experienced stress during ontogeny. Restrictions in skeletal growth may be revealed by comparing individuals experiencing stress to those with lower stress loads [15, 31]. Consequently, the skeleton represents a strong proxy for assessing developmental plasticity in response to stress.

Palaeopathologists may assess growth among children and adolescents as well as adult body size and shape phenotypes as indicators of past stresses experienced during ontogeny [32–34]. For example, researchers investigated if child growth varied across the 1200-year occupation of the Neolithic site of Çatalhöyük in Anatolia (Turkey) and if adult body sizes varied as well [35]. Children with indicators of physiological stress experienced developmental instability as indicated by smaller skeletal size-for-age during a time of population decline [35]. However, adult statures were normal relative to other Neolithic groups and overall growth patterns across ontogeny were comparable to modern children, suggesting that Çatalhöyük individuals experienced stress, but buffered against these challenges to maintain normal growth trajectories.

Palaeopathological researchers may use archaeological or historical information to infer potential stresses, and examine skeletal remains to assess how people responded to diverse challenges, including socioeconomic changes [36–38], environmental constraints [39] and infectious disease loads [40, 41]. For instance, Newman and Gowland [34] looked at 18th- and 19th-century London children and adolescents to see if social status affected growth. Historical information was used to identify four cemeteries encompassing different social status levels to see if high status children more effectively buffered the challenges of urban life. There were no differences among children from different social strata but all children fell behind a modern comparative group, indicating how challenging urban life was at this time. This particular study examined children and adolescents; however, in some cases only adult skeletal remains may be available to infer if constraints operated during earlier life stages [8]. Studies exclusively looking at adults may compare adult morphologies between groups with different archaeological or historical contexts, with reduced linear dimensions often interpreted as evidence of compromised growth [16]. Understanding factors that affect growth with implications for adult morphological variability may improve palaeopathological interpretations of skeletal size variation among past groups.

Clinicians will track whether an individual is following a normal linear growth trajectory and use this information to plan interventions and improve linear growth outcomes if faltering is observed [42]. Additionally, as per the Barker hypothesis and the Developmental Origins of Health and Disease (DOHaD) framework, early life constraints that impact growth may affect later life health, including cardiometabolic disease risk [29, 43–46]. For example, North Korean children who experienced early life nutritional deprivation resulting in reduced height-for-age or stunting had a lower rate of fat oxidation than children who did not experience stunting [44]. This lower rate of fat oxidation would predispose the stunted children to excess adiposity and obesity later in life [44]. As obesity is associated with cardiometabolic disease risk [43], this example demonstrates how early life constraints affecting growth may also shape adult health risks. This connection between growth and later life health provides further motivation to identify growth faltering among living children.

In this article, we seek to further bridge the gap between linear growth studies of living children and those of children recovered from bioarchaeological contexts. Until the 1990s, bioarchaeological research had largely excluded children [33], meaning that foundational work on the bioarchaeology of linear growth has some catching up to do. Palaeopathologists often incorporate perspectives from studies of living children; however, contemporary studies may not include the most appropriate children for comparison to global archaeological contexts. Interpretations in both biomedicine and palaeopathology could be improved by creating linear growth standards specific to bone measurements. By solely examining height, clinicians may be missing the opportunity to identify more nuanced instances of growth faltering that may predispose children to adverse adult health outcomes. Observations from palaeopathology may highlight stressors affecting linear growth by incorporating deep time perspectives that encompasses hundreds or thousands of years, which cannot be captured in contemporary human biology. Evolutionary perspectives on the origins of health and disease in children, including a better understanding of factors impacting child growth, would be of great benefit to clinical researchers. For example, studies examining child growth variation in past populations provides a basis for examining how variation in child body size and shape intersects with childhood obesity [47]. Public health bodies are concerned with the metabolic consequences of poverty, infectious diseases, and rapid changes in nutrition and lifestyle [48]. Such phenomena have taken place in past populations, and can be evaluated palaeopathologically, further connecting bioarchaeological evaluations of child growth and clinical interests today.

Here, we: review the methods currently used in both palaeopathological and biomedical research to evaluate linear growth trajectories among children and adolescents; identify new approaches that may harmonize methods across these fields; and identify future directions for research in both fields that may clarify how linear growth varies under diverse life circumstances.

## MEASURING GROWTH IN MODERN POPULATIONS

**Figure 1.**
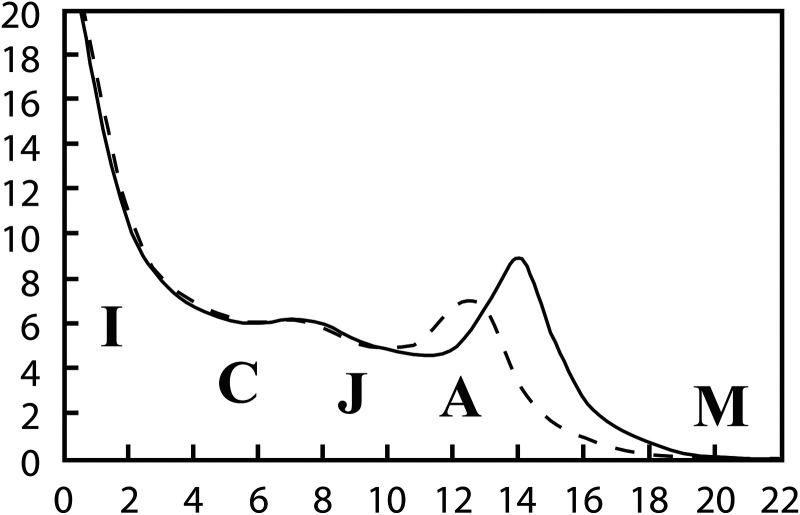
Velocity curves of growth in height for healthy girls (dashed lines) and healthy boys (solid lines) showing the postnatal stages of the pattern of human growth. Note the spurts in growth rate at mid-childhood and adolescence for both girls and boys. The stages of human postnatal growth are abbreviated as follows: I, infancy; C, childhood; J, juvenile; A, adolescence; M, mature adult. Original figure by Barry Bogin, modified by the authors [2]. Modified image created by V. Lukich

Human growth assessment allows clinicians to examine how environmental and genetic influences affect human body shape and size and represents a proxy for examining basic biological processes [49]. Many early studies identified a consistent pattern of postnatal linear skeletal growth in humans [50–53]. Broadly, during the infant phase (birth to three years), linear growth decelerates relative to the intrauterine phase, and during the childhood phase (three to seven years) growth continues at a steady rate [2] By the juvenile phase (seven to 11 years), growth decelerates only to increase again during the adolescence phase with the adolescent growth spurt (11–18 years) before slowing again after 18 years of age [2] (see Fig. 1). Other organ systems follow different trajectories during these phases. For example, reproductive organ growth only increases after approximately 11 years of age, whilst brain tissue growth decelerates around five years of age [2].

The predictability of linear child growth has allowed researchers to create normalized growth standards or growth reference data for clinical evaluations of child health. These standards are typically developed from longitudinal studies that track child growth across development. In the past, growth standards for different countries were produced in the form of centile charts [5]. A centile chart is a size-for-age chart that indicates whether a child is normal, above, or below normal size for their age Fig. 2 [54]. The lines that cross the plot are known as centiles, short for percentile, and indicate the percentage of population child growth in which an individual child falls. For example, if child A’s height falls along the 50th centile then 50% of children the same age are shorter than child A. Waterlow and colleagues [55] suggested the use of standard deviation scores (SD scores) rather than centiles as applying centile charts to comparatively deprived populations resulted in a large proportion of children falling below the lowest centile. An SD score is a normally distributed variable with a mean of zero and a standard deviation where values greater than zero indicate larger than average sizes and values less than zero indicate smaller than average sizes. A given individual is assigned a z-score that represents their distance from the population mean. This method enables linear growth charts to account for children who are either very small or very large for their age.

**Figure:**
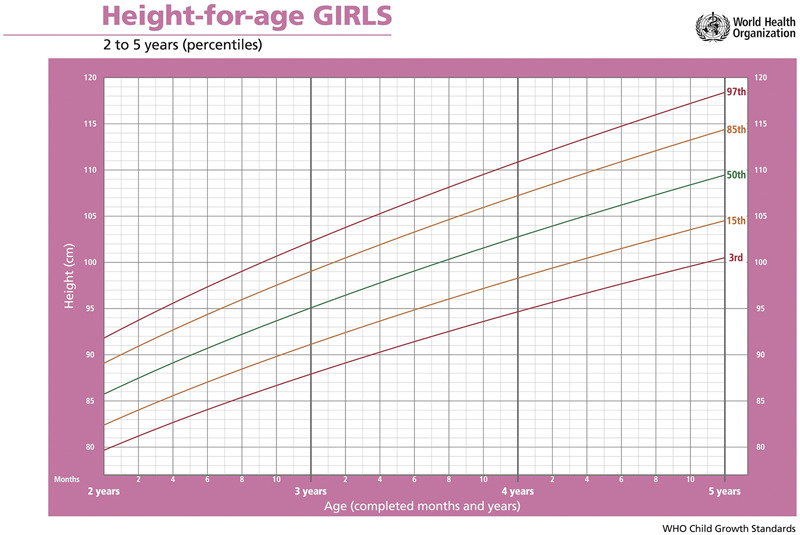


A range of measurements can be collected from children to quantify linear growth. Height (or stature) is measured to create an estimate of growth velocity, with the understanding that a normal growth trajectory in height typically indicates good general health [56]. Linear growth retardation (or linear growth faltering) is defined as a failure to reach one’s linear growth potential. Linear growth retardation implies that children are too short for their age, but does not imply that they are stunted [57]. Stunting is defined as having a height-for-age SD score < –2 standard deviations below the mean [58]. Although stunting rates have been decreasing, an estimated 21.3% (144 million) of children under 5 years of age globally experienced stunted growth in 2019 [59]. The value of height as a measure for growth velocity is based on accurate reporting of height measurement error and the principle that growth velocity estimates based on stature often fail to reflect previous growth or predict future growth [60, 61]. Weight is measured in growing children as a means of estimating nutritional intake and may also be assessed using weight-for-age SD scores. Body mass index (BMI), or the ratio between height and weight, may also be calculated to evaluate obesity risk [62]. These measures are all used to assess growth, but we will focus on height and other linear dimensions for the remainder of this article.

Measuring growth can be divided into three contexts: screening, surveillance or monitoring [42]. In screening, growth can be measured in a specific subset of a population with prescribed characteristics, such as children who are falling above or below a cut-off point for height. Children initially measured in screening may then be followed up in surveillance to assess the process of growth. Surveillance may identify a child with an unexpected pattern of growth, usually leading to the growth of this child being monitored clinically [42]. A child with shorter stature than expected for their age may have a growth hormone deficiency or conditions such as Turner’s syndrome or coeliac disease [63]. Stresses experienced early in life also affect growth outcomes, with the most significant effects occurring if stresses are experienced during the prenatal period, infancy and early childhood [64–66]. Catch-up growth during later childhood and adolescence may compensate for some early life growth restrictions [67, 68]. However, this process may be compromised among older children and adolescents if challenges are experienced during these later developmental stages or interventions are omitted [3, 68].

Comparisons of groups experiencing stress to growth standards or intra-population comparisons incorporating lifestyle information illustrate how growth may be compromised in different contexts. For example, height may vary in response to immune function [19], psychosocial and socioeconomic stressors [2, 20], and undernutrition [2, 63]. Such studies collectively indicate that, when compared to normal growth patterns, groups experiencing certain stressors may experience growth faltering during childhood and adolescence. Identification of these key stressors guide interventions to improve growth outcomes and inform new studies clarifying how growth disruptions arise in contemporary children.

## MEASURING GROWTH IN PAST POPULATIONS

Palaeopathologists use a range of techniques to assess linear child growth and factors that may have altered growth among past populations. When analyses are centred on adult human skeletal remains, standard osteometric methods are typically used to measure long bones directly [69]. Palaeopathological studies of children may measure diaphyseal lengths, or, for example, maximum long bone lengths when looking at older adolescents with fused epiphyses [70]. These measurements are used alongside age-at-death estimates and other osteological information to form an individual’s osteobiography, or the biological profile of an individual at the time of their death [69]. Additionally, researchers may assess skeletal indicators of specific diseases, such as rickets [71], and indicators of physiological stress, such as cribra orbitalia, porotic hyperostosis and linear enamel hypoplasias [34, 35, 40]. Indicators of physiological stress may identify stressful episodes in a child’s life and contextualize linear growth perturbations among past groups [35, 40]. This article will not address new approaches for interpreting indicators of physiological stress as linear growth assessments are our focus. However, we encourage researchers to continue investigating potential aetiologies of these indicators [72–75]. This work is essential to their continued use in identifying stressors that may have affected a child’s ability to follow a normal linear growth trajectory.

Palaeopathological assessments are unable to track linear growth across an individual’s childhood in a longitudinal fashion, as skeletal remains represent a ‘snapshot’ of a child’s status at their time of death. This makes it difficult to calculate growth velocity or create true growth curves [75]. Palaeopathological studies rely on cross-sectional analyses of children from different ages across childhood and adolescence to infer growth velocities. Growth curves, centiles and SD scores as used by clinicians may be compiled using cross-sectional information on linear dimensions and age-at-death estimates derived from dentition or epiphyseal fusion among adolescents to assess how linear growth occurred in a given population [76, 77]. While there may be limitations with age estimates based on dentition and epiphyseal fusion [78–82], these approaches allow researchers to identify typical linear growth patterns of children of unknown ages despite the limitations presented by bioarchaeological contexts.

Studies may combine osteometric information, indicators of physiological stress, and archaeological information to see if childhood skeletal dimensions were affected by external factors. Researchers may compare children with and without skeletal markers of specific diseases or indicators of physiological stress to see if linear growth varies between these groups [83–85]. Temporal comparisons may also clarify if growth patterns change over time in the face of, for example, socioeconomic transformations like the adoption of agriculture [86, 87]. Regional comparisons may identify if children who experienced diverse environmental conditions had different growth outcomes [38, 88]. For example, Bennike *et al*. [89] compared the long bone lengths of children and adolescents from medieval Danish burial grounds associated with a leprosarium to burial grounds associated with an Augustinian monastery, with the former representing individuals with a health and social disadvantages and the latter representing relatively privileged individuals. They found that the disadvantaged adolescents from the leprosarium burial grounds had shorter long bone lengths than the privileged adolescents from the monastery burial grounds. Comparisons between individuals or groups may also be based on estimates of stature or body mass derived from osteological measurements. Estimation formulae have largely been derived from modern studies of children and adolescents of predominantly Euro-American middle-to-upper class backgrounds with known age, sex and height [90, 91]. Skeletal remains of children and adolescents may not be available for investigating past linear growth trajectories. Adult skeletal dimensions may instead be used as proxies for early life linear growth experiences, with variation in the skeletal measurements of adults interpreted as the consequences of earlier growth perturbations [8]. Additionally, child growth may be plotted as a percentage of attained adult dimensions in intergroup comparisons to help account for potential genetic differences between groups [92].

Cross-sectional linear growth information from bioarchaeological contexts may be compared to contemporary longitudinal datasets based on the principle that modern children likely experienced normal growth trajectories and represent a proxy against which past children may be measured. As most growth standards, such as the WHO growth standards [93], are derived using living stature, there may be difficulties in comparing bioarchaeological information to these standards. Stature estimation formulae for children may be used to harmonize between past and modern contexts; however, this may introduce some error in the calculation of stature from skeletal element dimensions [94].

The limitation of comparing measurements of skeletal elements to living height measurements may be reduced using modern studies where skeletal measurements of long bones with fused and unfused epiphyses across the entire ontogenetic series are available [75, 95]. The most frequently used comparator that meets these criteria is the Maresh dataset [96–98]. This reference dataset is comprised of longitudinal measurements for upper and lower limb long bones from 2 months to 18 years of age taken from radiographs of approximately 200 modern healthy children of largely European descent from Colorado in the 20th century [96–99]. Additional information on anthropometry and nutrition were also collected.

As the Maresh dataset includes measurements of long bones with both fused and unfused epiphyses across a large developmental window, it has been extensively used as a comparator in bioarchaeology. Johnston [95] was one of the earliest studies to examine past linear growth in reference to the Maresh dataset to assess how environmental conditions affected growth at the Indian Knoll site in Kentucky, USA. In more recent years, this reference dataset has been used to assess past growth trajectories in a range of contexts [76, 100–103].

However, there are issues with the use of the Maresh dataset in palaeopathology. The Maresh dataset is focused on Euro-American children from middle to upper-middle class backgrounds, which may not be wholly representative of the growth patterns expected among past or contemporary populations [103, 104]. For example, few if any longitudinal childhood growth studies exist for Asian populations [105]. Palaeopathological comparisons in Asia could be done using Maresh [98]; however, it is not known whether Western reference data are appropriate for child growth in Asian populations [105]. Children in the Maresh study were noted to be larger and heavier than children from other North American growth studies [99]. Other datasets of long bone measurements are available that do encompass different socioeconomic and geographic contexts [106], and have been used in anthropological studies [107]. However, they do not necessarily encompass the full ontogenetic range, include all long bones, or incorporate as much detail regarding lifestyles.

Palaeopathological studies of growth often include cautions around interpretation. There are persistent questions about whether comparing those who did not survive the stresses of childhood and those who did presents an interpretive challenge [108, 109]. For example, Vercellotti and colleagues [37] found that taller statures could arise in bioarchaeology due to both favourable conditions during growth and greater environmental stresses leading to high selection and catch-up growth among survivors of early life stresses. Additionally, bioarchaeologists are frequently constrained by limited numbers of individuals available for study, issues of skeletal preservation, and sometimes limited archaeological information. Despite these limitations, however, methodological improvements allow for closer investigations of how external factors affect growth in these groups. Analyses of adolescent growth in medieval England provide new methods for estimating pubertal stage in human skeletal remains [100, 110]. This has allowed Lewis and colleagues [111] to demonstrate that chronic illnesses, malnutrition and environmental pollutants may have delayed pubertal development in past populations [111]. This study sets the stage for further investigations of how changes in pubertal timing may affect linear growth [112].

Biocultural information is limited in palaeopathology, but we can continue to focus on groups with clearer contextual narratives based on archaeological or historical data. An example of a recent study with a clear narrative is DeWitte’s [40] work on pre- and post-Black Death groups from 14th-century London. The Black Death epidemic represents a significant yet time-limited stressor. Historical sources indicate health was declining before the Black Death, potentially related to famine, other diseases and rural–urban migration. Health improved after the Black Death potentially due to increases in resource availability. DeWitte [40] examined tibial length among males and females from London cemeteries pre- and post-dating the Black Death. Male tibia lengths declined then improved in accordance with historically recorded changes in health conditions, while female tibia lengths declined post-Black Death as conditions seemingly improved. DeWitte and Lewis [112] found that earlier menarche among females, detected via skeletal indicators of pubertal stage [111, 112], may have occurred as conditions improved, resulting in earlier female growth cessation.

The above study reveals the nuance that can be achieved in palaeopathology by focusing on specific episodes in the past. This type of work could be readily compared to contemporary research with clinicians further exploring the relationship between menarcheal timing and bone growth longitudinally, allowing for an understanding of pubertal timing and linear growth that translates across centuries. Some palaeopathologists are advocating for stronger connections to clinical research to identify the factors that are most likely to impact skeletal growth to improve interpretations of past growth variability [37, 113]. It is imperative that novel, diverse comparative datasets for skeletal dimensions are created and biocultural impacts on bone tissue today are clarified, alongside ongoing palaeopathological investigations of child growth. In concert, these research avenues would connect growth patterns of past children to children growing in the present.

## BRIDGING MEASUREMENTS PAST AND PRESENT

Comparing the linear growth of living children to those from bioarchaeological contexts is challenging due to differences in sample composition, measurement techniques, biocultural detail, and issues with interpretations. However, such comparisons identify key factors affecting growth and development, refine interpretations regarding past groups, and may improve interventions for contemporary children. Here, we outline several approaches that may help bridge the gap between clinical and bioarchaeological skeletal growth research. Bioarchaeological contexts inherently limit the measurements that can be used to compare past and present children. However, contemporary medical imaging allows us to closely track skeletal growth among modern children in ways that complement bioarchaeological methods. We can address issues with the representativeness of current growth reference datasets by including a more diverse range of children in future studies. Palaeopathologists can collaborate with clinicians to develop datasets that incorporate survey questions designed to match the kinds of information available for past groups.

### Medical imaging and child growth

Researchers are increasingly repurposing medical imaging technologies, such as dual-energy X-ray absorptiometry (DXA), magnetic resonance imaging (MRI) and computed tomography (CT), to track skeletal growth. DXA is primarily a means of measuring bone mineral density via low-level X-ray beams, but can also be used to assess body composition or monitor chronic medical conditions like anorexia nervosa [114]. As the entire skeleton is visualized, DXA allows long bones, joint epiphyses, and skeletal elements indicative of body breadth to be observed in a single scan. DXA is more frequently used clinically for imaging children as it emits a lower radiation dose than CT. In addition, the open configuration of the scanner is reassuring to patients and their parents [115] and each scan takes less than 5 min to complete [116], both of which represent advantages over CT and MRI. These advantages place DXA as an ideal technology for creating bone-specific growth standards for living children.

DXA has been used to update growth standards and investigate the impact of different diseases on child growth (see Table 2; [117–140]). Linear measurements of skeletal elements have been collected from DXA scans of children. Abrahamyan and colleagues [141] collected humeral, radial, tibial and femoral length from whole body DXA scans of 413 Caucasian participants (170 boys and 243 girls) between 5.9 and 18 years of age in order to predict the normal length of the forearm or leg when planning hand or leg prosthetics. Völgyi and colleagues [142] collected radial, humeral, tibial and femoral lengths, as well as bi-iliac breadth and medio-lateral pelvic inlet breadth from whole body DXA scans of 396 Finnish girls between the ages of 10 and 18 years to assess the timing of peak growth velocity for body height, weight and width. DXA studies have already been applied to bioarchaeological questions. Pomeroy and colleagues [143] used DXA scans of contemporary adults from India to develop updated stature estimate equations for South Asian contexts. These tools can help researchers characterize the normal pattern of skeletal growth in children today.

**Table 2.** List of studies that have used dual-energy X-ray absorptiometry (DXA) to create growth standards for growing children, and studies that have used DXA to investigate growth patterns of children living with different diseases

Study citation	Study country/geographic region	Sample size and sex ratio or health status	Age range of sample	Study objective
[117]	Brazil	541 children (170 girls, 371 boys)	12–17 years	Present reference data of whole body lean mass (LM), lean mass index (LMI), appendicular lean mass (ALM) and fat mass
[118]	China	10 818 (5309 girls, 5509 boys)	3–18 years	Provide sex-specific bone mineral density reference values
[119]	China	12 790 (6219 girls, 6571 boys)	3–18 years	Develop body fat reference centiles for evaluating total body fat development and fat distribution
[120]	Denmark	101 (46 girls, 55 boys)	10–16 years	Study whether prenatal pesticide exposure was still associated with body fat content and distribution in the children at puberty and the potential impact of both maternal and child PON1 Q192R genotype.
[121]	Denmark	99 (49 girls, 50 boys)	3 years	Develop predictive equations for estimating fat-free mass from bioelectrical impedance and anthropometry using DXA as reference method.
[122]	Egypt	30 (18 girls, 12 boys)	7–15 years	Assess the effect of asthma and its therapy on bone mineral density
[123]	India	920 (440 girls, 480 boys)	5–17 years	Provide gender and age specific data on bone parameters and reference percentile curves for the assessment of bone status
[124]	India	334 girls and boys (167 living with beta Thalassemia major, 167 healthy controls)	3.6–18.8 years	Assess size corrected bone density and bone geometry
[125]	Italy	82 (40 girls, 42 boys)	5–30 years	Investigate the correlation between the severity of the clinical condition, bone status and body composition parameters in children and young adults with cystic fibrosis.
[126]	Korea	449 (232 girls, 217 boys)	5–20 years	Gain normal reference values and to evaluate gender differences in total and regional body composition changes according to age and pubertal development stage
[127]	Mexico	1659 (806 girls, 853 boys)	5–18 years	Provide reference values for relevant bone health variables for healthy Mexican children and adolescents
[128]	New Zealand	89 girls	4–5 years	Variability in body composition and subsequent longitudinal changes in absolute fat mass (kg) and relative adiposity (fat percentage)
[129]	New Zealand	96 (47 girls , 49 boys)	3–8 years	Compare parental assessments of child body weight status with BMI measurements
[130]	Samoa	42 (17 girls, 25 boys)	18.7–24.6 months	Examine body size and composition by genotype
[131]	South Africa	1036 (518 girls, 518 boys)	2–23 years	Examine whether the relationship between stunting at age 2 years and body composition at 23 years is mediated by adolescent body mass index and pubertal development.
[132]	Thailand	367 (193 girls, 174 boys)	5–18 years	Establish normative data of bone mineral density, bone mineral content, bone area and lean body mass for healthy Thai children and adolescents; aged 5–18 years and evaluate the relationships between bone mineral density versus age, sex, puberty, weight, height, calcium intake and the age of menarche.
[133]	United Kingdom	130 girls (13 girls with eating disorders, 117 healthy controls)	10–18 years	Assess body composition of young females with eating disorders involving substantial weight loss, relative to healthy controls.
[134]	United Kingdom	153 (96 girls, 57 boys)	5–21 years	Evaluate DXA against the four-component model in obese children and adolescents in both cross-sectional and longitudinal contexts
[135]	United Kingdom	1251	5–18 years	Evaluate gender and ethnic differences in percentage body fat in British schoolchildren
[136]	United Kingdom	442 (203 girls, 239 boys)	5–18 years	Provide UK-specific reference data for the Hologic QDR Discovery DXA scanners
[137]	United States of America	294 girls	6–17 years	Assess bone mass change during growth
[138]	United States of America	821 (427 girls, 394 boys)	5–18 years	Construct new reference curves for lateral distal femur bone mineral density
[139]	United States of America	783 (402 girls, 381 boys)	1 years	Examine the longitudinal associations of fruit juice intake in infancy with visceral adiposity in mid-childhood and early adolescence.
[140]	Zimbabwe	600 (300 girls, 300 boys)	8–16 years	Determine the impact of HIV on BMD and muscle function in peripubertal children on antiretroviral therapy.

In the first column from the left, the citation for the study is listed, the second column listing the country or geographic region in which the study was conducted, the third column listing the study sample size and sex ratio or health status (as appropriate for the study), the fourth column listing the age range of the sample and the fifth column outlining the study objective.

DXA may be used to create new bone-specific growth reference information to identify normal patterns of growth in contemporary children and explore different types of morphological assessments, such as intralimb proportions. Intralimb and interlimb proportions, which may be more nuanced indicators of early life linear growth restrictions [15], can be assessed in contemporary children using DXA and corroborated with survey information to assess how biocultural factors influence body shape. Paredes and colleagues [144] explain the value of comparing humeral and femoral lengths between samples to provide more nuanced information on stress that could impact growth, with lower limb length being more sensitive to stress relative to upper limb length. Measurements of more canalized skeletal components, like joint epiphyseal measurements among older adolescents and young adults [27], may also be explored in relation to biocultural variables and less canalized skeletal dimensions, such as diaphyses. Canalization patterns have not been explored extensively in children and younger adolescents [145], but epiphyses and metaphyses have the potential to reflect patterns of canalization as joint-proximate regions have greater constraints than diaphyseal regions in order to maintain joint function. These approaches may better reveal how the skeleton responds to environmental conditions and help clinicians identify children with subtle growth restrictions and risks for later life health issues. These measurements directly correlate to those taken in palaeopathology, allowing for comparisons between bioarchaeological and modern groups. Intralimb proportions have been explored in bioarchaeology [8, 146]; however, they are not commonly compared to reference information from living individuals who grew under known conditions. We encourage clinicians and palaeopathologists to consider incorporating these methods or their results when designing future research projects.

### Increased diversity of bone growth reference data

Traditional growth standards, like the Maresh dataset, contained limited diversity. Contemporary height growth standards incorporate children from a greater range of geographic and socioeconomic contexts to encompass a wider range of ontogenetic variation [26]. However, bone-specific growth standards are available for a limited diversity of modern children [96–98, 106, 147] As predominantly middle- to upper-class Euro-American children and adolescents are represented in these reference datasets, comparisons to other global populations past or present are inherently limited, potentially skewing interpretations and results [148] Ideally, regional-specific standards would be developed [148]. Additionally, as both contemporary and past populations faced nutritional and other socioeconomic challenges, greater investigation of bone dimensions among groups facing socioeconomic challenges would clarify the amount of skeletal variation that may arise under these circumstances. For example, the Birth to Twenty Plus (Bt20+) cohort study in Johannesburg, South Africa, conducted longitudinal investigations of height, weight, and BMI and determined the range of variation in growth distance, growth velocity, and absolute dimensions across sex and ethnicity among Black and White boys and girls [149]. White children were 5 cm taller than Black children during adolescence [149]. This study also calculated how much of the variance in growth timing and magnitude was explained by early childhood and maternal conditions, including socioeconomic status. Nyati and colleagues found that these factors accounted for between 19.2 and 52.3% of the variance observed in height, weight and BMI. These studies are informative but building on them using tools such as DXA to investigate skeletal linear dimensions and proportions would allow for deep time comparisons of growth variability. Researchers are working to include a greater range of childhood experiences in modern skeletal studies [74, 107]; however, there is room to grow in this research area.

Whilst researchers often seek to limit the variability within a study group to ascertain normal growth circumstances, we advocate for embracing the diversity of childhood experiences and contextualizing variability with a greater amount of biocultural information collected via survey or interview. By incorporating comprehensive lifestyle information into study surveys and moving beyond the creation of growth reference data based on healthy, wealthy children, clinical researchers can better understand how stressors such as malnutrition, psychosocial stress, socioeconomic status affect linear skeletal growth and body proportions. The creation of both population-specific bone growth standards for non-Western contexts and quantification of the range of variation that may arise under challenging childhood circumstances would greatly help palaeopathological researchers identify the most appropriate contemporary comparative group and see if growth variability observed in the past overlaps with the variability observed under present stressful conditions. Differences between children from different geographic regions or socioeconomic contexts may or may not be evident upon further investigation [104, 106, 147]; however, this requires confirmation. As palaeopathologists contextualize results from non-survivors, ontogenetic information on modern children from different circumstances may clarify the spectrum along which bone growth may occur and improve our sense of how wide this spectrum can be. We advocate for clinicians and contemporary growth researchers to engage in this type of research and characterize how population-specific skeletal growth deficits may arise as children face different types of challenges, be they nutritional, psychosocial or pathological.

### Adapting the collection of biocultural information

As we increasingly acknowledge that ‘normal’ patterns may incorporate biases [25], clinical researchers must continue to assess children with diverse life circumstances. By restructuring surveys to include questions that may align with variables evident in bioarchaeology or more open-ended questions, researchers may improve interpretations for clinicians and bioarchaeologists. For example, researchers could use a tool like DXA to look at bone growth longitudinally and include children from different socioeconomic backgrounds, rather than excluding children from certain statuses to avoid a potential confounding variable. Researchers could instead record socioeconomic and lifestyle information and assess if bone growth varies with socioeconomic status. Clinical studies will need to inform participants that secondary data analysis may be undertaken to be compliant with ethical protocols. However, such additional information may provide more nuance to contemporary research and allow for more direct comparisons with past contexts. For instance, contemporary research on socioeconomic status could be compared to results from Newman and Gowland [34] to see if socioeconomic status or urban conditions have a greater role in shaping growth outcomes today.

## GOING FORWARDS

Growth studies among living children and children recovered from bioarchaeological contexts have provided a wealth of information. These studies identified normal growth trajectories and the environmental factors that cause deviations from normal patterns. However, clinicians and palaeopathologists would both benefit from the creation of new bone-specific growth reference data. Tools such as DXA could be used to assess multiple aspects of skeletal growth longitudinally with a low risk posed to children or adolescents. Incorporating a greater amount of lifestyle information and a greater diversity of children in new studies allows for clarification of the factors that shape bone growth and expand the observed range of variation in growth patterns among living children. These approaches would allow researchers to better characterize growth and when it falters in modern children and may provide more applicable reference datasets for palaeopathologists. Palaeopathologists can use this information to focus on archaeological contexts where factors that affect skeletal growth in contemporary studies are known to vary between subpopulations to clarify interpretations.

We advocate for greater communication between those researching growth past and present. Palaeopathology and clinical research have the capacity to improve each other in meaningful ways and moving from siloed investigations of human biology is crucial to understanding the growing human body as an integrated whole. Projects that feature collaborations between these fields can improve investigations of how stressors moderate growth today; interpretations of how children may have negotiated stressors in the past; and outcomes for children growing in the future. We propose harmonizing methods of assessing skeletal linear growth across clinical practice and palaeopathology, allowing both specialties to expand and make more direct comparisons across human history and into the present.
